# Analysis of the "Institutions of Practical Medicine, Delivered in Lectures, by Jo. Bapt. Burserius de Kanilfeld"

**Published:** 1799-11

**Authors:** 


### *Analysis of the "Institutions of Practical Medicine, delivered in Lectures, by* JO. BAPT. BURSERIUS DE KANILFELD," in Latin, 4 Vol. 8vo. Leipzig, 1798.

WE have just received a copy of the last edition of this truly valuable, and hitherto, in England, scarce work. We are sorry to observe, that the author did not live to complete his design, for the fourth volume, which was published after his death, concludes with the diseases of the abdomen. But before we commence any account of the work itself, we shall gratify our readers with such information as we have been able to procure respecting the medical character of the author. BURSERIUS was born at Trent, of an honourable family, and received his first rudiments of medicine from Felix Perger. He studied at the University of Padua for some time, and then removed to Bologna, where he devoted himself to the study of medicine under Laghi, Azzoguidi, Laurenti, Beccaria, and others. Here his progress was so rapid, that Bartholom Beccaria conceived the most flattering expectations of his future eminence; these expectations were more than realized. By the advice of Beccaria he settled as a physician in Fayence, where he continued to practise near twenty years, with every honourable mark of the approbation of his fellow citizens, and many princes of the sacred Roman empire, as, BARNI, CRESCENTI, SERRA, BOLOGNETTI, ODDI, STUPPANIO, BORROMEO, and the Popes CLEMENT XIII. and XIV. the former of whom distinguished him by many honourable privileges, and the latter appointed him Professor of Medicine in the restored University of Ferrara. In this situation it is incredible what numbers of patients applied for his advice, both personally and by letter, not only from the adjacent but the most distant states of Italy, and even how many physicians of great reputation requested to meet him in consultations. By these means, and the frequent mention made made of him by medical authors, his fame increased daily, till at length Charles Firmian, Embassador to the Queen Theresia, resolving to restore the celebrity of the University of Pavia, which had long fallen into neglect, invited Burserius by very honourable proposals to deliver public lectures on Chemistry and the Practice of Medicine, and also clinical lectures on the cases in the hospital, for the improvement of the pupils. With what exertion and with what success he discharged these important duties, no Italian is ignorant; not only his pupils, who came from the most distant countries for the sake of enjoying the advantage of his instructions, but also his professorial colleagues, who were much attached to him on account of his learning, elegant manners, and the candour of his mind, bore the most honourable testimony to his worth. The College of Physicians of Pavia elected him an honorary member of their body; the whole state and province were witnesses. But why should we dwell upon these external evidences of his high reputation? his own institutions on fevers, and other general diseases, afford the most decisive proofs of his merit.

During the ten years in which he discharged the duties of his Professorship, he was thrice unanimously elected Principal of the University, to the advancement and glory of which he contributed no less by his discipline than his learning. At length the Queen Theresia determined to commit the restoration of the health of her children Ferdinand and Beatrice Atestia to his care. This honourable appointment obliged him to leave the University of Pavia, where his loss was universally regretted, in order to reside at Milan, for the sake of being near the sick Princes. His anxiety night and day in the discharge of this duty contributed very materially to injure his own declining health, which the heart-felt thanks and princely gratitude of his royal patients and their parents, who imputed their recovery to his skill, could not restore. He resided seven years at court, caressed by his patrons, and universally esteemed and admired. He died Dec. 19, 1785, at the age of 61, of an abscess in the right kidney; the bladder was also said to be scirrhous, and the adjacent parts considerably diseased. His funeral, than which nothing could be more splendid or honourable, was personally attended by the young Princes. The loss of so great a man was severely felt by the whole city; for he was not more admired on account of his learning, than beloved for his polished manners and affability; so that he truly seemed to have been formed by nature to conciliate and secure the affections of all that knew him.

From the Introduction to the first volume, we give the following abridgement:

abridgement. "Medicine is properly enough divided into Theory and Practice." The former teaches thofe branches which may be deemed preparatory, and lays the foundations of the fcience; the latter comprifes the whole art, and completes the fuperftructure.

The Theory is employed in delivering and explaining the general doctrines of anatomy, phyfiology, pathology, femeiotice, hygiene, and therapeutics, together with an enumeration of the *Inftrumenta Medicinæ*, drawn from the three kingdoms of nature. And becaufe this delivers the firft rudiments of the art, and in every well regulated Univerfity, or fchool of medicine, is taught before the Practice, it has obtained the name of the *Inftitutions of Medicine.* How can it be expected that any one fhould conceive accurately of difeafe, its caufes and effects, preferve health by a proper regimen, or reftore it when loft, which are the objects of the practice of phfiyc, unlefs he poffefs a competent knowledge of the animal fabric, the organization, powers, faculties and functions of life and health? When this introductory knowledge has been acquired, the path to the more ufeful and valuable part of medicine, diftinguifhed by the title of *Practice*, becomes direct, plain, and eafy. It is the bufinefs of this part to deliver the hiftory, and explain the nature of every *genus* and *fpecies* of difeafe; to inveftigate their manyfold origen, to characterize their regular, peculiar, and diagnoftic marks; to point out the prognofis; and, laftly, to lay down the moft expedient indications of cure, deduced from reafon and experience. By thefe fteps we come to the bedfide of the patient, called Clinical Practice, to which three branches are particularly fubfervient, Dietetics, Surgery, and Pharmacy, according as diet, operations, or medicines, may be indicated.

Of thefe two branches of the Science of Medicine, I propofe to confine myfelf to the Practice folely, and to begin with FEVERS, not only as conftituting the moft common clafs of difeafes, and being very often Idiopathic, but alfo moft frequently combined with local affections. I am not infenfible of the difficulty of the tafk I undertake, and perhaps have not fufficiently weighed

*Noftri quid valeant humeri, quid ferre recufent.*

For the more authors I confulted who had written on Fevers, the more was I involved in obfcurity; fome were for reducing the whole number to a few genera, others endeavoured to extend them by divifions and fubdivifions, in a manner that baffled the memory and puzzled the underftanding. This fubject gave me no fmall trouble, but did not entirely difcourage me. I determined to procure all the beft authors I could find on on the subject of fever, and read them diligently once or twice, constantly noting the circumstances in which they agreed and in which they differed, as well as the causes of their difference. When I had continued this labour for several years, I then determined to try these opinions of the most celebrated authors by the Lydian stone of experience, in order to distinguish fact from opinion or hypothesis. Whatever therefore I have acquired by many years reading, meditation, and experience, that I believe may be relied on, is comprised in this volume, for the benefit of students in this science. I have not been biased by the tenets of any sect, nor even very solicitous *whence* doctrines or opinions came, but what they were, and by what evidence supported. *Formulæ*, or compositions of medicines, are rarely given, for many reasons, but chiefly that young men may not be induced to employ more time in copying *formulæ*, and committing them to memory, than in distinguishing diseases, and discovering indications, without which they must practise like empirics, to the disgrace of their profession. For it is the duty of a rational practitioner, having maturely weighed and settled the indications, judiciously to accommodate his simple medicines to them; or if compound forms should be necessary, he ought to mix and combine such as are adapted, not only to the particular disease and its causes, but to the age, sex, temperament, climate, season of the year, and other necessary circumstances: nor will this be very difficult for him to perform, who has stored his memory with the nature and powers of medicines, the forms of prescribing them, and the diet of the sick, all which are taught in the general doctrines of Therapeia or Pharmaceutics, which I suppose to have been previously acquired by the student.

With respect to the style, I have always preferred the plain and perspicuous, to the pompous, affected, or figurative, which I think very ill accommodated to the business of teaching. Ornamental periods, therefore, calculated to please the ear only, I have unreluctantly relinquished to those who are ambitious of the name of orators; for in teaching and explaining practical arts, we may say,

"*Ornari res ipsa negat contenta doceri.*"

It is a problem of no very obvious solution, to determine where a teacher of the practice of medicine ought to begin. The most usual mode is to begin with Fevers. This appears to us like a person commencing a course of lectures on astronomy, with the construction of tables for calculating the motions of the moon, or a course of chemistry with the analysis of minerals.

Burserius appears to have been fensible of this folecifm, and therefore introduces his work with a *Differtation* on Inflammation.

This arrangement appears to us highly judicious; for it is fcarcely poffible to treat of any fever, or even chronic difeafe, without frequent mention of Inflammation. He thus introduces the hiftory of it, which we have confiderably abridged. " When any part of the body becomes preternaturally warm, red, tenfe, fwelled, painful, or throbbing, it is faid to be inflamed. The *proximate caufe* of Inflammation is extremely obfcure, and the inveftigation of it has engaged the attention of medical authors from the earlieft ages of the fcience."

Hippocrates, in the book on wounds of the head, fays, *The parts furrounding an ulcer are inflamed, and become tumified by the influx of blood.* His difciples, however, either forgetting the influx of blood, or not believing it, imagined an acrid or glutinous determination to the part inflamed, and fometimes a pituitous and vifcid defluxion on the part.

Thefe opinions prevailed till Erafiftratus imputed inflammation and its attendant fever to a transfufion of blood into thofe veffels which are naturally deftined to circulate air. For the ancients imagined that the *arteries*, as being generally found void of blood after death, were defigned during health to circulate *air*, inftead of blood. This feems to have been the origin of the Boerhaavian *error loci*, as a caufe of inflammation.

The next innovators were, Galen, Oribafius, Ætius, Paulus Ægineta, and the other fupporters of the Galenic fyftem, who made inflammation to confift in a determination of hot blood to the part affected, which *diftilled* through the pores, and produced fwelling, pain, and rednefs; nay, Oribafius contended, that a putrefcent ftate of that fluid fo determined, was the caufe of the heat. But as foon as the chemical fect began to rival the Galenic, Willis fuggefted a *febrile effervefcence* of the blood as the caufe of phlegmonous inflammation; and the *alkalis* of Sylvius, his cotemporary, tended as little to illuminate this obfcure fubject. Such were the firft feeble attempts to fhake off the trammels of the Galenic fchool. Ettmuller, apparently at different times, advanced two opinions on this fubject: in the firft, he confiders the *increafed heat* as the principal circumftance to be inveftigated; and in order to judge of the quantity or degree of increafe as well as its caufe, he inftitutes an inquiry into the caufe of animal heat in general, during life and health, health, and concludes, that it arises from the mutual action of a volatile aerial acid in its natural state and its proper alkali upon each other. But the increased heat of inflammation he derives from a different source, viz. an influx of spirit or gas, which increases the mutual action of the acid and alkali to a morbid degree. He therefore does not hesitate to consider the accumulation of blood in the inflamed part as an accessory symptom, not as the cause of the heat. For he says, the pain arising from the increased action of the acid and alkali produces a contraction of the fibres, hence the diameter of the veins is diminished, the return of the blood impeded, which produces stagnation, accumulation, and inflammation.

His other opinion was, that a congestion of blood in the capillary veins and cellular membranes produced heat and pain. The material proximate cause, therefore, of inflammation, he defines to be, blood collecting and stagnating in any part, on account of the incapacity of the veins to transmit it from the arteries as fast as it arrives. The other symptoms he endeavours to explain as follows, viz. Since the blood which causes inflammation is red, spirituous, and hot, wherever it accumulates there must be an increase of redness and heat; and since more blood flows into the part than can return by the veins, swelling must take place, the nervous fibres must be distended, hence pain and all the symptoms of inflammation.

SYDENHAM, who very seldom relies on the theoretical speculations of others, but is generally his own ætiologist, taking his causes from what he deemed observation and experience, believed he had discovered *a peculiar diathesis, or state of the fluids*, in inflammation; and he speaks of it as consisting in a conflagration and fervour of the blood itself. He is not solicitous to enquire whether its motion be accelerated or retarded in any part. The blood, however, being thus heated and effervescent, and carried to all parts of the body, he thinks some of its inflamed and heated particles are one while applied to the brain, sometimes to the pleura, or lungs, sometimes to the skin, and being there deposited produce phrenitis, pleuritis, pneumonia, or erysipelas. This translation, or deposition of inflammation, says BURSERIUS, may often take place in acute fevers, and I would grant also in most inflammations which arise without any antecedent disease; but if we investigate the matter more closely, we shall not find the same account of their origin applicable to all kinds, for frequently the inflammatory diathesis of the blood is only a consequence, not a cause of the disease.

We shall now take a view of their notions, who referred every thing to to the laws of mechanics and hydraulics. Among these, Bellini is chief, who assumes the heat of the blood with the Galenists, but adds an *obstruction* in the capillary vessels. Pitcairn, of the same sect, thought the *obstruction* alone, sufficient to account for all the phœnomena of inflammation.

F. Hoffman not only admitted this kind of obstruction in the arteries which carry red blood, but also in the serous, the lymphatic, and the veins. Hence he deduces the following explanation of symptoms, viz. "The blood constantly flowing through the semiobstructed or constricted channels, must move with greater celerity; but being also deprived of its free passage, a part regurgitates to the larger branches, and there produces a more frequent diastole and systole of the arteries; whence arises a great attrition of the sulphureous parts, and extreme heat, which is distressing in proportion to the sensibility of the part affected."

Boerhaave made no improvements in the doctrine of inflammation, but Gorter, his pupil, saw sufficient cause for being dissatisfied with the explanation given by the mechanical physicians. He observed the increased pulsation of the arteries, which he concluded must arise from increased arterial action, and not from the obstruction of a few neighbouring branches. This argument he supported by hydraulic and anatomical experiments, as well as mathematical calculations; and he infers, that the proximate cause of inflammation, is a more vigorous *vital action* in particular branches of an artery, by which the red blood is impelled into the serous arteries, as appears in the albuginea of the eye.

That general inflammation, or inflammatory fever, is the effect of *increased action*, not only in a few branches of an artery, but in the whole arterial system. The increased tonic motion of the vessels which the Stahlians, (in conjunction with the *vis naturæ medicatrix*, or directrix of all vital actions) considered as the efficient cause of inflammation, appears very similar to the increased vital action of Gorter. Sauvages enlists under the Stahlian banners; but the single experiment of Haller on the separated heart of a frog, proves that muscular action is not governed by the mind, or sentient principle alone.

These, says *Burserius*, are the principal and most celebrated notions on the proximate cause of inflmmation, delivered by our predecessors, and from whence some other slight variations which we have omitted are obviously taken. Several opinions of our cotemporaries, such as a fermentation tation of the oleaginous parts of the blood; an increafed coagulability of the fibrous parts; a redundance of phlogifton or caloric; we pafs over as expofed to objections no lefs formidable.

Our author then proceeds to deliver his own opinion, p. 24, which we fhall endeavour to exhibit in as narrow a compafs as poffible.

It is agreed on all hands, fays he, that an inflamed part is red, hot, fwelled, painful, and affected with an unufual pulfation; fo that we may fafely conclude an unufual determination of blood to the part muft attend inflammation. But if all the blood carried thither by the arteries were as readily returned by the veins, I could not expect local inflammation to take place. In order, therefore, to produce inflammation, fwelling, heat and pain in any part, one of thefe two things muft follow; either the blood carried by the arteries is not entirely received by the veins, which may happen from various caufes; or that it is carried thither with a force fufficient to diftend and dilate the orifices of the lateral branches, or what are called the inorganic pores, and fo form to itfelf new channels. In either way the influent blood will fill and diftend the *vaja minima*; nay, thofe which are believed to admit only a fingle red globule at a time, now receive feveral together; they are enlarged, and acquire an unfual rednefs: nor is it an uncommon thing for the blood to be forced, or to ooze through the exhalent arteries into the cellular membrane, as Galen fuppofed, and as diffections have abundantly fhewn: nor does it feem improbable that the blood may be accumulated in the *ferous arteries*, fo as to prefs upon the adjacent veffels, and produce the obftruction fuppofed by Hoffman and Gorter.

The rednefs, tenfion, and fwelling of the part, proceed from the dilatation and repletion of the *vafa minima*, as well as the effufion of blood into the cellular membrane; and the increafed pulfation of the arteries, from the increafed velocity and quantity of blood determined to the part affected. As to the heat and fenfe of burning which attend inflammation, it is not to be expected that it fhould be fufficiently explained, till phyfiologifts have difcovered the true caufe of animal heat.

We may, however, at prefent venture to affert, that it depends upon the *quantity* of red blood, its accumulation, increafed motion, attrition, change of vafcular ftructure, or evolution of caloric in confequence of change.—While thefe changes are taking place, the nervous filaments muft neceffarily be diftended and divellicated, before they can adapt themfelves themselves to the new organization. Hence must arise an infinite diversity of pain, lancinating, shooting, gnawing, &c. according to the degree of inflammation, and the seat of the disease. Violent or continued pain, introduces spasm, whence the hard vibrating pulse so frequently observed to accompany inflammation.

[ To be Continued. ]

